# Optimization of Printing Parameters for Self-Lubricating Polymeric Materials Fabricated via Fused Deposition Modelling

**DOI:** 10.3390/polym17101401

**Published:** 2025-05-20

**Authors:** Peiyang Zhang, Feiyang He, Muhammad Khan

**Affiliations:** Centre for Life-Cycle Engineering and Management, Faculty of Applied Engineering and Science, Cranfield University, Bedford MK43 0AL, UKfeiyang.he@cranfield.ac.uk (F.H.)

**Keywords:** 3D printing, self-lubricating, coefficient of friction, infill density, printing speed

## Abstract

This study investigated the feasibility of fabricating self-lubrication material using fused deposition modelling (FDM) technology, focusing on the influence of printing parameters on tribological performance. Experiments were conducted using PA and ABS materials, with varying printing speed, infill density, and layer height across four levels. The research established regression equations and fitted curves to describe the relationship between printing parameters and the coefficient of friction (CoF). Validation experiments demonstrated the reliability of the models, with errors within 10%. The results indicate that reducing printing speed and increasing infill density enhance surface quality, with infill density exerting a more significant effect. The influence of layer height on surface quality depends on the printer characteristics, making precise quantification challenging. Additionally, this study confirms that resin-based samples produced via FDM exhibit self-lubricating potential. These findings contribute to the optimization of FDM-printed structures by balancing surface quality and tribological performance.

## 1. Introduction

Additive manufacturing (AM) has become a key technology in modern engineering, offering significant advantages in designing and producing complex components across various industries [1,2,3]. In the medical field, AM, particularly 3D printing, has shown great potential for creating customized implants that meet the specific needs of individual patients [4,5]. Among the different 3D printing techniques, fused deposition modelling (FDM) is widely used due to its cost-effectiveness, ease of use, and compatibility with a range of thermoplastic materials [6,7]. However, the performance of FDM-printed parts, especially in biomedical applications, is highly dependent on the selection of printing parameters. Achieving optimal tribological properties, such as low friction and wear resistance, is critical for developing self-lubricating implants that can function effectively in the human body.

Porous self-lubricating materials have been extensively studied for their ability to reduce friction and wear [8,9,10]. These materials are typically created by forming a porous structure and then impregnating it with lubricants. For example, Cao et al. [11] used a rotational sintering method to produce porous self-lubricating materials from ultra-high molecular weight polyethylene. They found that sintering at 180–190 °C for 10 min with a load of 3.6–3.8 g resulted in a stable coefficient of friction (CoF) of 0.1. Similarly, Samyn et al. [12] incorporated graphite powder into polyimide to create solid self-lubricating materials, which showed significantly reduced wear compared to pure polyimide. Li et al. [13] further demonstrated the effectiveness of porous structures by using spark plasma sintering to fabricate NiTi alloys with varying porosity. After oil impregnation, the CoF of these alloys dropped from 0.391 to 0.041. Despite these advancements, the relationship between pore characteristics (e.g., size, distribution, and porosity) and the material’s mechanical properties remains unclear, limiting the application of these materials in biomedical implants.

The tribological performance of FDM-printed components exhibits complex dependence on processing parameters, as evidenced by recent parametric studies [14,15,16]. For instance, Frunzaverde et al. [17] studied the effect of printing temperature and filament colour on the CoF of PLA samples. They found that the lowest CoF was achieved at a printing temperature of 220 °C. Wang et al. [18] investigated the tribological properties of polyamide (PA) samples printed at temperatures ranging from 240 °C to 260 °C and observed that the CoF reached a minimum at 245 °C. In addition to temperature, infill patterns and density also play a crucial role. Renganathan et al. [19] compared the CoFs of PLA samples printed with three different infill patterns (Concentric, Linear, and Hilbert) and found that the infill pattern significantly influenced friction behaviour. Karthick et al. [20] further demonstrated that increasing the infill density from 60% to 100% reduced the CoF of polypropylene parts.

Layer height is another critical parameter in FDM printing. Amirruddin et al. [21] studied the effect of layer height and raster angle on the friction force of printed parts and found that larger layer heights generally resulted in lower CoFs. Similarly, He et al. used an orthogonal experimental method to optimize the layer height, nozzle temperature, and printing speed for PEEK materials. Their results showed that the best tribological performance was achieved at a layer height of 0.2 mm. However, the interaction between layer height and other printing parameters, such as nozzle temperature and infill density, requires further investigation to understand its full impact on friction and wear.

In addition to fused deposition modelling (FDM), other AM techniques have also been explored for fabricating self-lubricating materials. Selective Laser Sintering (SLS) has been employed to produce functional components exhibiting wear resistance and chemical durability using materials such as iglide^®^ i9-ESD [22]. Similarly, self-lubricating composites fabricated via Digital Light Processing (DLP), including PTFE-filled polyimide structures [23], have demonstrated the potential to improve tribological performance.

While these studies consider the effects of individual printing parameters, most research has focused on isolated factors rather than the combined effects of multiple parameters. Additionally, there is limited research on the self-lubricating properties of FDM-printed structures, particularly how printing parameters influence these properties. Although Fu et al. [24] explored the use of coatings to enhance the self-lubricating performance of implants, the intrinsic self-lubricating behaviour of the printed materials themselves has not been thoroughly investigated.

To address these gaps, this study aims to systematically investigate the effects of key FDM printing parameters (e.g., nozzle temperature, infill density, and layer height) on the tribological performance of FDM polymers. The goal is to identify the optimal combination of parameters for producing self-lubricating surfaces with low friction and high wear resistance. Furthermore, this study will develop a predictive model to describe the relationship between printing parameters and tribological performance, providing a practical framework for designing and manufacturing customized parts with enhanced functionality and longevity.

## 2. Materials and Methods

The primary aim of this study was to investigate the impact of various printing parameters on the tribological performance of the fabricated components. Tribological testing was carried out to collect the friction properties data and develop an empirical model.

### 2.1. Design of Experiment

During the 3D printing process, various printing parameters influence the quality of the fabricated components, including layer height, extrusion temperature, printing speed, infill density, material selection, and printing path [17,18,25,26,27]. As reviewed in the Introduction, the existing literature suggests that temperature has a relatively minor effect on the surface CoF. Consequently, this study primarily focuses on examining the influence of the remaining parameters on tribological performance.

A Design of Experiments (DoE) methodology was employed to systematically analyse the effects of independent variables on the frictional behaviour of the printed components. A full factorial DoE approach was considered, where each parameter was assigned multiple levels to assess its impact comprehensively.

The coefficient of friction (CoF) on the surface of printed components is inherently linked to print quality, which is significantly affected by printing speed, infill density, and layer height. Therefore, these three parameters were selected as independent variables to evaluate their influence on surface tribological properties. Additionally, material composition plays a crucial role in determining frictional behaviour. For comparative analysis, acrylonitrile butadiene styrene (ABS) and nylon (PA 12) were chosen as the experimental materials. Four distinct levels were assigned to each for the three selected printing parameters, resulting in a total of 128 unique experimental conditions. After fabrication, the printed specimens’ surface friction characteristics were evaluated through experiments. The detailed experimental design matrix is presented in Table 1.

### 2.2. Specimen Preparation

**Figure 1:**
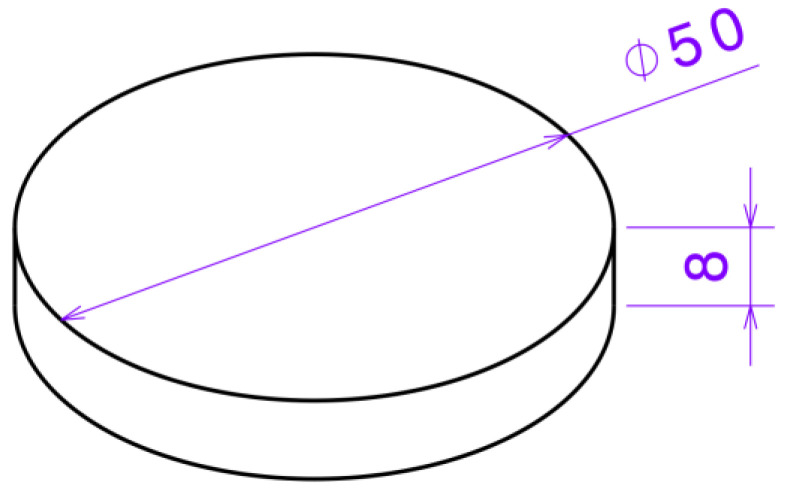
Specimen dimensions.

A CAD model, depicted in Figure 1, was designed for the printing experiment. The specimen is a cylindrical structure with a diameter of 50 mm and a height of 8 mm, which meets the fixture requirements of the Anton Paar TRB^3^ tribometer (Anton Paar (Shanghai) Trading Co., Ltd., Shanghai, China) used in friction testing. It was modelled using UGNX v41.0 software.

According to the experimental design outlined in Table 1, a total of 128 distinct samples were fabricated for testing. The slicing process was conducted using Ultimaker Cura 5.1.1 software with standard settings. The infill pattern followed the default ±45° alternating cross-hatch. The nozzle diameter was 0.4 mm. For ABS, the nozzle and bed temperatures were set to 235 °C and 90 °C, respectively; for PA12, they were set to 260 °C and 100 °C. A single-variable testing approach was strictly followed throughout the experiment, ensuring that all parameters remained constant except for the variables under investigation. The G-code generated during the slicing process was employed to execute the printing on the Ultimaker 2+ printer. During the printing process, two filament materials, ABS and PA12 from Ultimaker [28,29], were utilized to produce the required experimental specimens with varying printing parameters. The selection of ABS and PA12 was based on both practical considerations and scientific rationale. These two thermoplastic materials are among the most commonly used in FDM due to their availability, compatibility with standard printers, and well-documented processing characteristics. Importantly, they also exhibit markedly different tribological behaviours, which makes them suitable candidates for comparative analysis in self-lubricating applications [30]. However, it is acknowledged that the results obtained in this study may be material-specific. Further investigations will be necessary to determine whether the observed trends can be generalized to other thermoplastics used in FDM.

**Figure 2:**
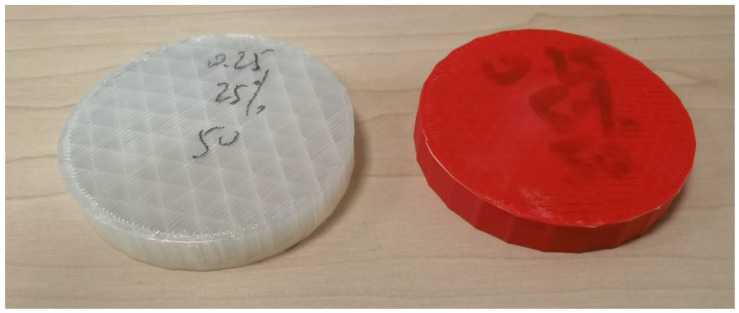
Two printed test samples. Left: PA12. Right: ABS.

It is worth noting that only one specimen was printed for each parameter combination. To minimise variability, all samples were fabricated using the same printer and filament batch under controlled indoor conditions. Although minor fluctuations in ambient temperature and humidity may exist, their effects are negligible compared to the extrusion temperature (over 230 °C) and are unlikely to influence the specimen quality significantly. All specimens were printed using a consistent bed temperature of 90 °C, as recommended by the material manufacturer for ABS and PA12. After each print, the build plate was allowed to cool naturally to room temperature before removing the specimen. The plate was then reheated to the target temperature before initiating the next print. This thermal cycling procedure was followed to prevent residual heat accumulation at the build location and to ensure stable thermal boundary conditions for each specimen. Future work will include repeated prints and randomized sample placement to enhance statistical validity. The final printed specimens are presented in Figure 2.

### 2.3. Experimental Setup and Procedures

An Anton Paar TRB^3^ tribometer was utilized to measure the surface coefficient of friction. Before initiating the experiments, it was calibrated to ensure measurement accuracy. Each specimen was then securely fixed using a three-jaw chuck. A small eccentric stainless-steel ball (hardness 223 HB, Young’s modulus 194 GPa) was mounted on the tribometer arm and positioned perpendicular to the disc surface. A constant normal load of 5 N was applied to each specimen during testing.

The experiments were conducted with wear track radii of 10 mm and 15 mm while the disc rotated at a constant speed of 250 rpm, and the average of the two measurements was calculated to determine the coefficient of friction for the sample surface. The tests were performed at room temperature (25 °C), with each friction experiment lasting one minute. To prevent contamination and ensure consistency, the contact surfaces were thoroughly cleaned with ethanol after each test to remove any residual resin material from the stainless-steel ball. All friction experiments were conducted following this standardized procedure. This approach ensured reliable and representative data for each specimen.

### 2.4. Data Processing and Modelling

The tribometer measured the CoF at 2 Hz sample frequency. According to the manufacturer, the instrument provides a friction force resolution of 0.06 mN with a standard deviation of less than 0.0025 mN [31]. The corresponding uncertainty in the CoF measurement was estimated to be below ±0.0005. During each test, the CoF was recorded continuously as a function of time. As expected, the CoF fluctuated during the initial transient period and gradually stabilised. To minimise the influence of transient fluctuations, the average CoF value within the 5–10 s interval (10 repeated test values) after the onset of sliding was used as the representative value for analysis.

The Spearman correlation coefficient is employed as a nonparametric measure of association to assess the correlation between the printing parameters and the coefficient of friction. Subsequently, this study employs polynomial functions for curve fitting to accurately determine the relationships between variables and assess the goodness of fit of the regression model via R^2^ and Root Mean Square Error (RMSE).

## 3. Results and Discussion

**Figure 3:**
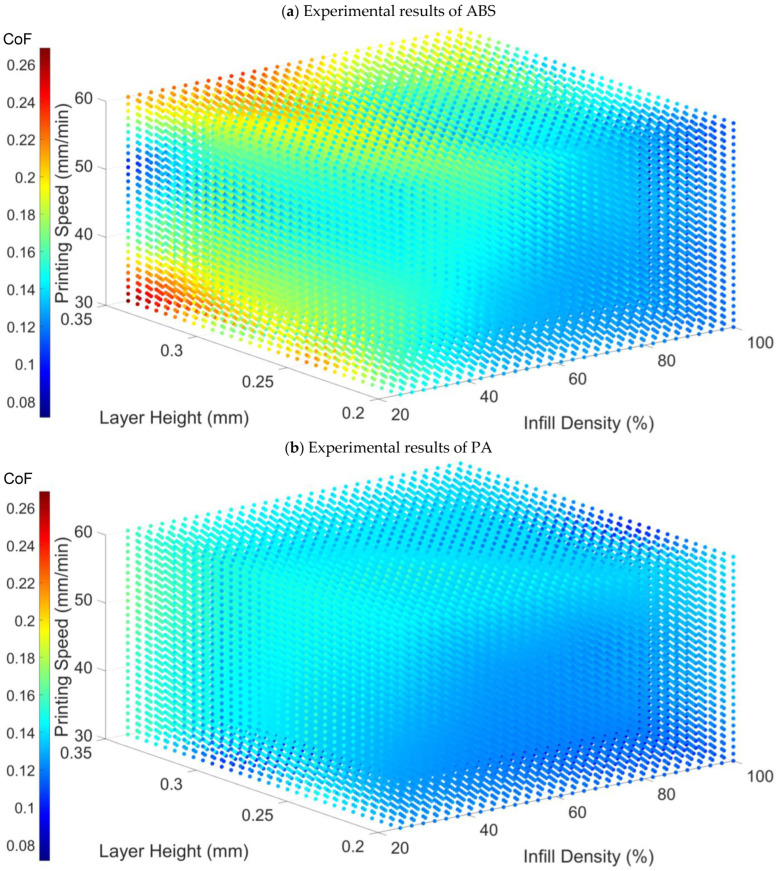
Experimental results of (**a**) ABS. (**b**) PA. The colour scale represents the CoF.

The detailed CoF results are presented in Appendix A. Data visualization was performed using interpolated 3D point clouds, where colour variations were used to illustrate changes in the CoF. The axes represent the variations in three independent printing parameters, as depicted in Figure 3.

Through the visualization of data graphs and the measured data, it can be observed that the CoF increases with the increase in printing speed; conversely, it decreases with the increase in infill density. In addition, the CoF of ABS is higher than that of PA. However, the relationship between layer height and the coefficient of friction is nonlinear, and the direct relationship between the two cannot be discerned from the measured data. Further derivation of this relationship requires subsequent equation fitting.

While increasing infill density and reducing printing speed contribute to lower CoFs, practical considerations must be considered during fabrication. For instance, setting the printing speed to 60 mm/s may lead to excessive acceleration, inducing nozzle vibrations that compromise print precision. Additionally, when printing ABS with a layer height of 0.35 mm, the significant material shrinkage during cooling, coupled with the high layer height, can result in warping and surface irregularities, such as burr formation on the surface.

### 3.1. Influence of Layer Height on CoF

**Figure 4:**
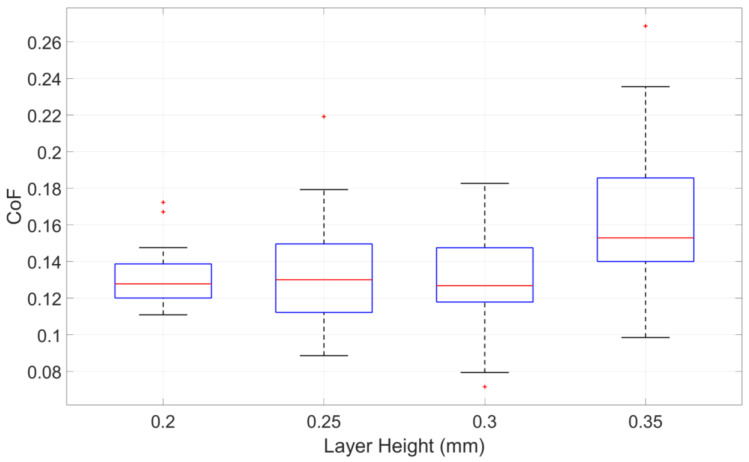
Influence of layer height on CoF, based on combined data from ABS and PA specimens.

To quantitatively analyse the effect of layer height on the CoF, the average values of all experimental data for each fixed layer height are summarized in Table 2. The boxplot for the average CoF of different layer heights is shown in Figure 4.

As presented in Table 2, the effect of layer height on the CoF differs between the two materials in the lower layer height range. For ABS, the CoF increases steadily with layer height, while PA shows a minimum at 0.30 mm, followed by a sharp rise. Despite these differences, both materials exhibit a marked increase in CoF at 0.35 mm, suggesting a common threshold beyond which surface quality or interlayer adhesion deteriorates. These observations indicate that the influence of layer height is partially material-dependent, particularly at intermediate levels. Figure 4 presents the combined trend across both materials and shows the general impact of layer height.

Based on the observations, no clear linear correlation between layer height and the CoF was identified. This finding aligns with the conclusions drawn by Amirruddin et al. [21]. The absence of a direct linear trend may be attributed to multiple factors related to the printing process. In particular, the noticeable increase in CoF observed at a layer height of 0.35 mm can likely be explained by a substantial decline in print quality when using the small-scale printer employed in this study. The deterioration in surface uniformity at this height may have contributed to the increased surface quality, subsequently leading to higher friction values.

The influence of layer height on friction behaviour can be understood by considering key factors such as interlayer adhesion and overall surface quality. A larger layer height generally increases surface irregularities, which may enhance friction due to uneven contact between the printed specimen and the tribometer. Conversely, smaller layer heights typically improve interlayer adhesion, leading to greater structural integrity and consistency, which could influence frictional properties. Additionally, reducing layer height tends to enhance surface smoothness, potentially lowering the CoF by minimising asperities that contribute to friction.

**Figure 5:**
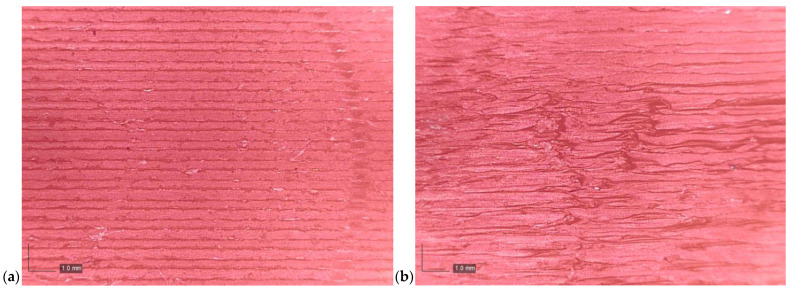
Microscope images showing surface morphology at different layer heights: (**a**) 0.20 mm and (**b**) 0.35 mm.

To further investigate the relationship between layer height and surface quality, a Dino-Lite AM4113T digital optical microscopy was employed to observe the surface morphology of selected specimens. As illustrated in Figure 5, the images reveal that specimens printed at a layer height of 0.35 mm exhibit more pronounced surface irregularities compared to the sample printed at a 0.2 mm layer height. The nozzle may not sufficiently compress the extruded material at larger layer heights, resulting in decreased surface quality and void formation. This factor contributes to poorer surface uniformity and higher measured CoF values.

Ultimately, the influence of layer height on the CoF was less significant compared to printing speed and infill density. The observed nonlinear trends hint at the potential of tests to optimize layer height alongside other printing parameters. Future research may explore multi-factor interactions in more complex use cases.

### 3.2. Influence of Infill Density on CoF

**Figure 6:**
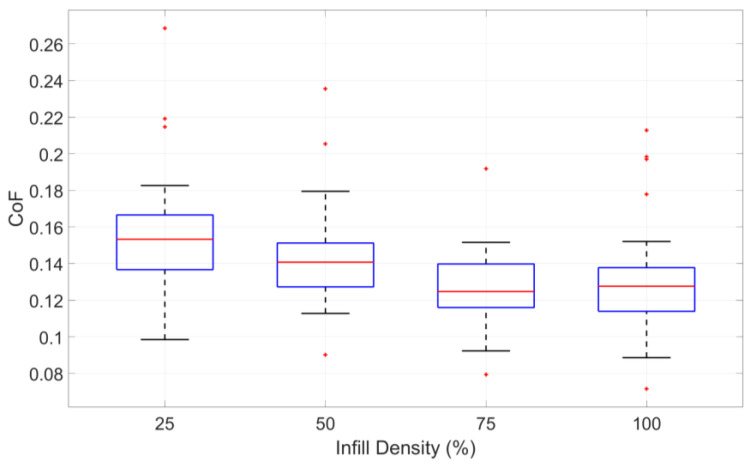
Influence of infill density on CoF, based on combined data from ABS and PA specimens.

The data for all specimens with the same infill density were averaged, and the corresponding results are summarized in Table 3. To further illustrate the distribution and variability of the CoF across different infill densities, a boxplot representation is provided in Figure 6.

The data presented in Table 3 indicate a clear trend where the CoF decreases as infill density increases. However, beyond an infill density of 75%, the CoF stabilises, showing minimal variation. This behaviour suggests that, while lower infill densities contribute to increased surface interactions and higher friction, further densification beyond a certain threshold has a negligible impact on tribological performance. Notably, both ABS and PA exhibit similar trends in response to infill density. This trend is also visually represented in Figure 6, where the data distribution confirms the decreased effect at higher infill densities.

This finding is consistent with previous studies by Srinivasan [32] and Karthick [20]. This relationship can be attributed to several key factors related to the mechanical properties of the printed specimens. One of the primary reasons for this trend is the increase in surface hardness associated with higher infill densities. A denser internal structure enhances the material’s resistance to deformation, thereby reducing frictional forces. For instance, a specimen with 70% infill density exhibits a lower CoF than one with 30% infill density, demonstrating the correlation between material density and tribological performance.

Additionally, higher infill density increases the effective material contact area and internal support structure, leading to a more uniform load distribution and reduced friction and wear. The enhanced structural integrity resulting from greater infill density also contributes to improved durability and resistance to surface deformation, further reducing the CoF. In summary, increasing infill density enhances surface hardness, material contact area, and structural strength, all contributing to a reduction in surface roughness and, consequently, the coefficient of friction.

However, once the infill density surpasses a certain threshold, such as 75% in this experiment, the benefits plateau as the material’s surface hardness and structural strength reach optimal levels. This phenomenon can be attributed to the fact that the internal structure achieves sufficient stiffness and support at higher infill densities, minimising deformation during sliding contact. Consequently, further increases in infill density have a limited effect on the overall tribological behaviour. Minor fluctuations in CoF at high infill levels may instead be dominated by localized factors such as melt-bonding quality between adjacent filaments, variations in layer adhesion, and subtle differences in the printing path. These microstructural influences warrant further investigation through detailed microscopic and mechanical analyses.

### 3.3. Influence of Printing Speed on CoF

**Figure 7:**
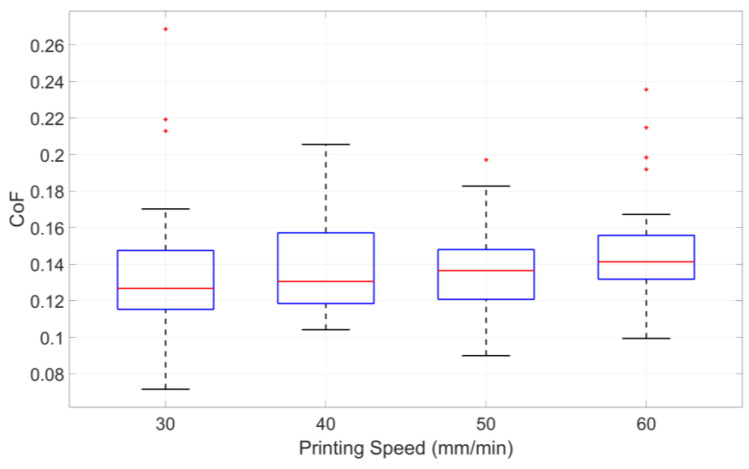
Influence of printing speed on CoF, based on combined data from ABS and PA specimens.

The experimental data corresponding to each print speed were averaged to evaluate the influence of print speed on the CoF, with the results presented in Table 4. Additionally, the variability and distribution of CoF values across different print speeds are visually depicted in the boxplot shown in Figure 7.

The data presented in Table 4 show that increasing print speed generally corresponds to a decline in surface quality and a tendency toward higher CoF values, particularly for PA. However, the relationship is not strictly monotonic for ABS, and some fluctuations are observed. This relationship suggests that higher print speeds may introduce surface irregularities that contribute to increased friction. The same trend is further validated through the visualized data in Figure 7.

This observation aligns with the findings of Sujithra [13]. Several factors may contribute to this phenomenon. Higher print speeds can result in poor interlayer adhesion, leading to lower surface quality and compromised structural integrity. Additionally, rapid extrusion may cause inconsistent material flow, reducing the time available for proper deposition and affecting surface smoothness. Thermal management is another critical factor, as higher print speeds reduce cooling time, which can lead to deformation and surface defects, further increasing roughness. Moreover, excessive speed can introduce machine vibrations and positioning errors, negatively impacting print precision and overall surface quality.

While increasing print speed reduces fabrication time, it also shortens cooling intervals and can potentially enhance surface uniformity. However, excessively high speeds may introduce mechanical instabilities that degrade print quality. Furthermore, a comparative analysis of print speed and infill density reveals that infill density has a more pronounced influence on the CoF. Among these two parameters, infill density plays a more significant role in determining surface tribological behaviour. The subsequent equation fitting and modelling analysis will further explore this distinction.

### 3.4. Correlation Coefficients Results

The correlation coefficients between the independent variables and the CoF were analysed based on the data presented in Appendix A. The experimental results confirm that the relationship between printing parameters and the CoF is nonlinear. The correlation coefficient results obtained are summarized in Table 5.

The results indicate that all three parameters—layer height, infill density, and printing speed—exhibit measurable correlations with CoF, although their relative strengths vary by material. Notably, layer height shows a moderate correlation but is not included directly as an independent variable in the regression models.

Instead, the data were stratified by layer height to control for its influence and reduce potential parameter interactions. Four polynomial regression models were developed for each material under fixed layer height conditions (0.20 mm, 0.25 mm, 0.30 mm, and 0.35 mm).

This stratified modelling approach enabled a more precise analysis of how infill density and printing speed jointly affect CoF. Given the nonlinear trends observed in the experimental data and the interdependent nature of the printing parameters, polynomial functions were employed to capture the complex relationships effectively.

### 3.5. CoF Prediction Model and Validation

#### 3.5.1. Prediction Model Development

The corresponding regression equations are summarized in Table 6. Here, x represents printing speed, y denotes infill density, and z corresponds to the coefficient of friction (CoF).

Furthermore, the regression equations considering layer thickness as a variable were obtained by processing the 64 data sets for each material. These equations comprehensively consider three key variables: printing speed (*x*), infill density (*y*), and layer height (z). The CoF (a) results are as follows:

For ABS material:

$$a=-0.33+1.17y+6.54z+0.0302y^{2}-11.2z^{2}+0.0096xy-0.056xz-20.0yz+0.0102xyz+56.3yz^{2}+13.2y^{3}z-0.24xz^{3}-0.00088x^{2}yz-0.043xy^{2}z-1.27y^{4}+0.024x^{2}z^{2}$$

R^2 = 0.66127 and RMSE = 0.020116

For PA material:

$$a=-0.964+0.557y+8.98z+0.0001x^{2}+0.543y^{2}-16.7z^{2}-0.0199xy-0.038xz+0.00048x^{2}y-7.71y^{2}z-0.000012x^{3}z+2.09y^{3}z+30.1yz^{3}+0.12xy^{2}z-0.28xyz^{2}-0.00038x^{2}y^{2}+0.0031x^{2}z^{2}$$

R^2 = 0.62031 and RMSE = 0.02006

When all three parameters—printing speed, infill density, and layer height—are considered simultaneously, the regression model exhibits a lower R^2^ value (approximately 0.65) compared to models with a fixed layer height. This reduction in goodness-of-fit can be attributed to two main factors. First, the combined influence of multiple parameters introduces more complex nonlinear interactions that are difficult to capture with low-degree polynomial functions fully. Second, the experimental data itself contains inherent scatter, which arises from small variations in printing conditions, material inconsistencies, and measurement uncertainties. These factors collectively contribute to increased variability, thereby reducing the predictive accuracy of the overall model.

For self-lubricating performance, the CoF must be maintained at or below 0.2 [33]. Based on the regression equations derived in this study, the PA material meets this requirement under all tested conditions. For the ABS material, self-lubricating performance can be achieved when the layer height is below 0.32 mm, the printing speed is less than 54 mm/s, and the infill density exceeds 26%.

The modelling results indicate that, when the layer height was fixed, the R^2^ consistently exceeded 0.80, while the RMSE remained below 0.015, suggesting that the model’s predictive accuracy was relatively high. However, when all independent variables were considered simultaneously, the R^2^ value decreased to approximately 0.65. This reduction in predictive accuracy may be attributed to the increased complexity of the model and the limitations of the current dataset, which may not be sufficient to capture the interactions among multiple parameters fully. Additionally, the model exhibited poor predictive performance for ABS specimens printed with a layer height of 0.35 mm. This is likely due to the lower surface roughness at this layer height, which may have introduced experimental inconsistencies and contributed to deviations in the results.

It is acknowledged that the fitted model is limited to the specific materials and printing platform used in this study. The predicted parameter values are theoretical outputs of the regression and may not be directly achievable on other FDM systems, where layer height and speed are typically constrained to discrete values. Nevertheless, the model provides useful guidance in identifying optimal parameter windows and understanding the relative influence of each input variable.

#### 3.5.2. Model Validation

The reliability of the fitted regression equations was evaluated through experimental validation. The detailed printing parameters used in the validation process are listed in Table 7.

**Figure 8:**
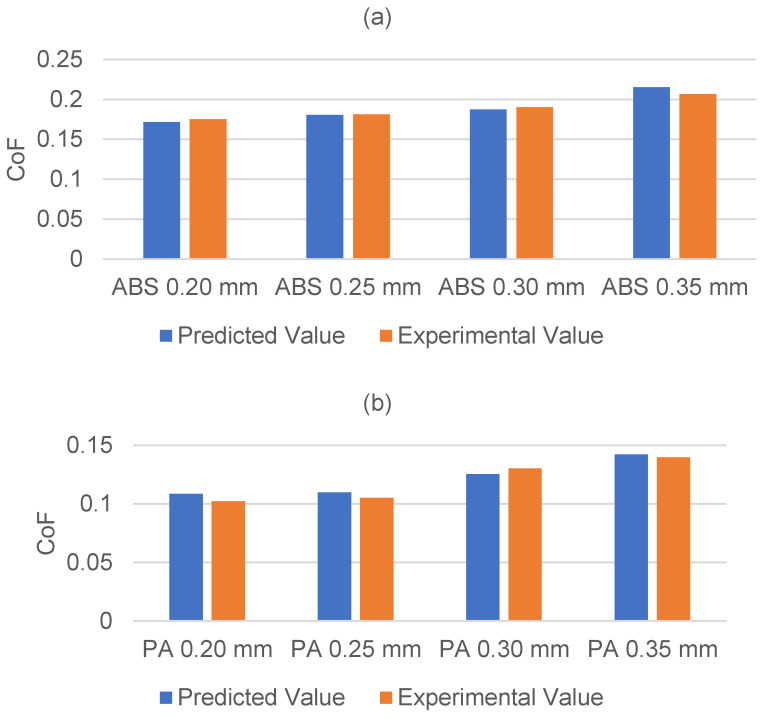
Validation experiment CoF results for (**a**) ABS, and (**b**) PA.

To ensure consistency, all specimens were fabricated using the same 3D printer and printing materials as in the initial experiments. The surface CoF of the printed specimens was then measured using the tribometer under identical testing conditions. The discrepancies between the experimentally measured values and the values predicted by the regression equations are illustrated in Figure 8.

The data indicate that the discrepancy between the experimentally measured values and the predicted values from the regression model remains within 10%. This level of accuracy further confirms the reliability of the modelled relationship between the printing parameters and the CoF, validating the effectiveness of the proposed regression equations.

## 4. Conclusions

This study systematically examined the influence of key printing parameters—printing speed, layer height, and infill density—on the surface friction performance of FDM-printed structures using ABS and PA materials. A key contribution is its comprehensive investigation of the combined effects of multiple printing parameters.

The results indicate that increasing printing speed leads to a higher CoF. In contrast, increasing infill density lowers the CoF. The impact of layer height on CoF is nonlinear, as it depends on the specific interaction between print and surface quality. While moderate layer heights contribute to smoother surfaces, excessively high layer heights (e.g., 0.35 mm for ABS) result in surface defects that significantly increase friction.

This research also developed validated regression models as predictive tools for selecting printing parameters to achieve desirable tribological performance. The findings suggest that for PA, self-lubrication can be achieved across all the tested conditions, whereas for ABS, self-lubricating behaviour is observed when the layer height is below 0.32 mm, the printing speed does not exceed 54 mm/s, and the infill density is greater than 26%. Further studies will include detailed surface roughness measurements and dimensional analysis to better understand the mechanisms underlying frictional behaviour in FDM-printed materials.

## Figures and Tables

**Table 1:** Parameters considered in the experiment.

Parameter	Unit	Levels			
		1	2	3	4
Layer thickness	mm	0.20	0.25	0.30	0.35
Infill density	%	25	50	75	100
Printing speed	mm/s	30	40	50	60
Material	/	ABS	PA12	/	/

**Table 2:** Average CoF for specimens with the same layer height.

	0.20 mm	0.25 mm	0.30 mm	0.35 mm
PA	0.1277	0.1263	0.1134	0.1562
ABS	0.1341	0.1361	0.1471	0.1875

**Table 3:** Average CoF for specimens with the same infill density.

	25%	50%	75%	100%
PA	0.1423	0.1297	0.1203	0.1213
ABS	0.1727	0.1562	0.1377	0.1383

**Table 4:** Average CoF for specimens with the same printing speed.

	30 mm/s	40 mm/s	50 mm/s	60 mm/s
PA	0.1155	0.1279	0.1332	0.1369
ABS	0.1495	0.1468	0.1507	0.1579

**Table 5:** Results of Spearman correlation coefficient.

	ABS	PA
Infill density	−0.460	−0.392
Layer height	0.452	0.313
Printing speed	0.080	0.359

**Table 6:** R^2^, RMSE and regression equation for different layer heights.

Material and Layer Height	R2	RMSE	Regression Equation
ABS 0.20 mm	0.8782	0.0131	$z=0.0144+1.66y-0.0000769x^{2}-2.6362y^{2}-0.05033xy+0.103xy^{2}-0.0596xy^{3}+1.2243y^{2}$
ABS 0.25 mm	0.9700	0.0087	$z=0.0155+0.024y-0.000027x^{2}+0.0552xy+0.0029xy^{2}$
ABS 0.30 mm	0.8749	0.0103	$z=0.304-1.03y-0.00026x^{2}+0.339y^{2}+0.0633xy-0.0766xy^{2}+0.0383xy^{3}$
ABS 0.35 mm	0.7210	0.0240	$z=-1.7107+0.0868x+6.47y-0.00091x^{2}-4.58y^{2}-0.308xy+0.00326x^{2}y+0.218xy^{2}-0.0023x^{2}y^{2}$
PA 0.20 mm	0.9166	0.0047	$z=-0.083+0.0211x+0.3156y-0.000355x^{2}+0.505y^{2}+0.00133x^{2}y+0.052xy^{3}-0.947y^{4}-0.00115x^{2}y^{2}$
PA 0.25 mm	0.8290	0.0155	$z=1.6833-0.0547x-4.35y+0.000516x^{2}+2.93y^{2}+0.1469xy-0.0014x^{2}y-0.0864xy^{2}-0.011xy^{3}+0.00094x^{2}y^{2}$
PA 0.30 mm	0.9883	0.0032	$z=0.1452+0.0931y+0.0001x^{2}-0.00976xy+0.00573xy^{3}$
PA 0.35 mm	0.9223	0.0044	$z=0.9598-2.90y-0.00093x^{2}+4.56y^{2}+0.0040x^{2}y-0.276xy^{2}-0.00005x^{3}y+0.0521xy^{3}+0.00000000162x^{4}+0.00167x^{2}y^{2}$

**Table 7:** Setting parameters for validation experiment.

Test No.	Material	Infill Density	Printing Speed	Layer Height
No. 1	PA	60%	45 mm/s	0.20 mm
No. 2	PA	60%	45 mm/s	0.25 mm
No. 3	PA	60%	45 mm/s	0.30 mm
No. 4	PA	60%	45 mm/s	0.35 mm
No. 5	ABS	60%	45 mm/s	0.20 mm
No. 6	ABS	60%	45 mm/s	0.25 mm
No. 7	ABS	60%	45 mm/s	0.30 mm
No. 8	ABS	60%	45 mm/s	0.35 mm

## Data Availability

Data is contained within the article. Dataset available on request from the authors.

## Appendix A

**Table A1:** Experimental results of CoF.

Material: ABS		Infill Density			
Layer Height (mm)	Printing Speed (mm/s)	25%	50%	75%	100%
0.20	30	0.1476	0.1278	0.1256	0.1152
0.20	40	0.1723	0.1312	0.1232	0.1162
0.20	50	0.1434	0.1472	0.1349	0.1206
0.20	60	0.1358	0.1672	0.1278	0.1109
0.25	30	0.2191	0.1318	0.1248	0.1175
0.25	40	0.1793	0.1426	0.1172	0.1064
0.25	50	0.1519	0.1353	0.107	0.0899
0.25	60	0.1456	0.1324	0.1247	0.1521
0.30	30	0.1702	0.1483	0.1245	0.1198
0.30	40	0.1623	0.1592	0.1516	0.1154
0.30	50	0.1827	0.1542	0.1385	0.1377
0.30	60	0.1638	0.1446	0.1468	0.1341
0.35	30	0.2687	0.1574	0.1409	0.2128
0.35	40	0.1571	0.2055	0.1313	0.1779
0.35	50	0.0985	0.1795	0.1126	0.1971
0.35	60	0.2148	0.2355	0.1918	0.1984
**Material: PA**		**Infill Density**			
**Layer Height (mm)**	**Printing Speed (mm/s)**	**25%**	**50%**	**75%**	**100%**
0.20	30	0.1216	0.1176	0.1152	0.1127
0.20	40	0.1267	0.1199	0.1202	0.1298
0.20	50	0.1376	0.1251	0.1198	0.1324
0.20	60	0.1453	0.1423	0.1398	0.1374
0.25	30	0.1473	0.1283	0.0923	0.0886
0.25	40	0.1573	0.1128	0.1258	0.1116
0.25	50	0.1543	0.1393	0.1037	0.1325
0.25	60	0.1523	0.1592	0.1168	0.0993
0.30	30	0.1062	0.0902	0.0794	0.0715
0.30	40	0.1245	0.1196	0.1041	0.1161
0.30	50	0.1268	0.1208	0.1142	0.1255
0.30	60	0.1324	0.1268	0.1245	0.1311
0.35	30	0.1592	0.1452	0.1398	0.1328
0.35	40	0.1571	0.1427	0.1409	0.1379
0.35	50	0.1694	0.1392	0.1487	0.1425
0.35	60	0.1594	0.1454	0.1402	0.1386
